# Physical Activity, Watching Television, and the Risk of Obesity in Students, Texas, 2004-2005

**Published:** 2011-04-15

**Authors:** Adriana Pérez, Deanna M. Hoelscher, Andrew E. Springer, H. Shelton Brown, Steven H. Kelder, Cristina S. Barroso, Brian C. Castrucci

**Affiliations:** The University of Texas Health Science Center at Houston, School of Public Health, Austin Regional Campus; The University of Texas Health Science Center at Houston, School of Public Health and Michael and Susan Dell Center for the Advancement of Healthy Living, Austin, Texas; The University of Texas Health Science Center at Houston, School of Public Health, Austin, Texas; The University of Texas Health Science Center at Houston, School of Public Health, Austin, Texas; The University of Texas Health Science Center at Houston, School of Public Health, Austin, Texas; The University of Texas Health Science Center at Houston, School of Public Health, Brownsville, Texas; Georgia Department of Community Health, Atlanta, Georgia

## Abstract

**Introduction:**

The epidemic of childhood obesity has been well-documented. Prevalence of obesity among students in Texas is higher than the US prevalence. Our objective was to understand the combined influence of physical activity and television viewing on weight status of students in Texas.

**Methods:**

Students in grades 4, 8, and 11 participated in the School Physical Activity and Nutrition survey during the 2004-2005 academic year. Multinomial logistic regression tested the associations between both being overweight and obese (vs underweight/normal weight) and the combined influence of physical activity and watching television, adjusting for age, grade, race/ethnicity, language spoken at home, and percentage of economically disadvantaged students in the school. We used 5 physical activity indicators to describe students' physical activity.

**Results:**

Girls who participated in less than 3 days of exercise per week to strengthen or tone muscles and watched 2 hours or less per day of television had increased odds of being obese (adjusted odds ratio, 1.8; 95% confidence interval, 1.1-3.0) compared with girls who participated in 3 or more days per week of exercise to strengthen or tone muscles and watched 2 hours or less per day of television. Boys in our study who watched 3 or more hours per day of television and did not meet physical activity recommendations had increased odds of being obese in all of our 5 physical activity indicators.

**Conclusion:**

Although results varied by physical activity indicator and sex, our findings provide further evidence for the combined effect of high television watching and low physical activity engagement on the risk for obesity in children and adolescents.

## Introduction

Current US estimates from 2003 through 2006 indicate that the prevalence of obesity was 17% for children (aged 6-11) and 18% for adolescents (aged 12-19) (1). The prevalence of obesity among students in grades 4, 8, and 11 in Texas (20% in 2000-2002 and 2004-2005) is higher than the US prevalence (2-4). Despite US recommendations to promote regular physical activity (5) and reduce television viewing (6), obesity continues to be a serious health issue in the United States.

Physical activity is essential for health at any age (5). Guidelines for Americans recommend that children and adolescents engage in at least 60 minutes of physical activity daily (5). During the time of our study, *Healthy People 2010* (7) set a goal for adolescents to engage in moderate physical activity for at least 30 minutes on 5 or more of the previous 7 days or in vigorous physical activity that promotes cardiorespiratory fitness 3 or more days per week for 20 or more minutes per occasion. In 2003, the Centers for Disease Control and Prevention (CDC) reported that, among 9th-grade students, 25% of boys and 35% of girls did not engage in regular physical activity (8). Similarly, statistics on 11th-grade students showed that 26% of boys and 39% of girls did not engage in regular physical activity (8). According to the latest CDC Youth Risk Behavior Survey (YRBS) (9), 35% of US high school students watched television for 3 or more hours on an average school day with no statistically significant changes since 2003.

Although associations between physical activity and obesity and between watching television and obesity have been well-documented separately (10,11), the combined influence of physical activity and television viewing on obesity is still subject to debate (12). The purpose of this article is to explore the association of the combined influence of physical activity and television watching on weight status among Texas public school students, where the prevalence of obesity is high (3). We hypothesized that children and adolescents who reported not meeting physical activity recommendations and watching television for 3 or more hours per day would have increased odds of being obese or overweight.

## Methods

During the 2004-2005 school year, we conducted the School Physical Activity and Nutrition (SPAN) survey. This validated survey allowed us to monitor the prevalence of child and adolescent weight status, physical activity habits, dietary intake, meal patterns, and nutrition knowledge (13,14). A full description of the 2004-2005 SPAN study design and its participants is available elsewhere (15). Briefly, SPAN targets a single grade to represent each developmental level of school children: 4th grade for elementary, 8th grade for middle, and 11th grade for high school. Depending on school district and school research recommendations, we obtained either passive or active informed consent. Informed assent was also obtained from all participating children and adolescents. The Committee for the Protection of Human Subjects at the University of Texas Health Science Center at Houston (HSC-SPH-00-056), the institutional review board of the Texas Department of State Health Services (04-062), and participating school districts approved this study.

We used school and school district information from the Texas Education Agency (TEA) for the 2003-2004 school year to create the sampling frame for this study. A total of 3,863 schools constituted the sampling frame. SPAN used a multistage probability-based study design. Developed sampling weights and poststratification adjustment factors provided state-level representative data by sex and major racial/ethnic groups (African American, Hispanic, and white/other). The sample for 4th-grade students was 7,907 with a grade population of 248,838; for 8th grade, 8,827 and 291,672; for 11th grade, 6,456 and 233,753, for a total of 23,190 participants. Response rates were 96% for 4th grade, 95% for 8th grade, and 93% for 11th grade.

We used "What language do you use with your parents most of the time (English, Spanish, or other)?" as a proxy for acculturation. The percentage of economically disadvantaged students within the school was our proxy for students' economic status. TEA calculates the percentage of economically disadvantaged as the sum of the students eligible for free or reduced-price lunch or for other public assistance divided by the total number of students in a particular school times 100. We used standardized procedures to directly measure all students' height and weight. We calculated body mass index (BMI) as weight in kilograms divided by the square of the height in meters. Using growth charts from CDC to calculate BMI for sex and age (16), we classified students into underweight/normal (<85th percentile), overweight (≥85th percentile to <95th percentile), and obese (≥95th percentile) weight status categories.

We assessed 5 recommended physical activity indicators (17). First, we examined participation in regular physical activity (RPA), defined as engaging in vigorous physical activity that made the respondent sweat and breathe hard for at least 20 minutes on 3 or more of the past 7 days, or moderate physical activity that did not cause the respondent to sweat or breathe hard for at least 30 minutes on 5 or more of the past 7 days. This variable was based on the US recommendation for physical activity at the time of the study (8). We dichotomized regular physical activity into *met* or* did not meet recommended levels of physical activity*. The second indicator was participation in organized physical activities (OPA) or taking lessons such as martial arts, dance, gymnastics, or tennis. The third indicator was participation in exercises to strengthen or tone muscles (EST) on 3 or more of the past 7 days (for students in 8th and 11th grades only). We asked "On how many days of the past 7 days did you do exercises to strengthen or tone your muscles, such as push-ups, sit-ups, or weight lifting?" and then collapsed the number of days for this indicator. The fourth indicator was participation in physical education (PE) classes on 4 or more days during an average school week. We asked "In an average week when you are in school, on how many days do you go to physical education?" and then collapsed the number of days for this indicator. The fifth indicator was participation in the past year in 1 or more sports teams (ST) run by the school. For 4th-grade students, the sports teams question was "During the past 12 months, on how many sports teams did you play?" compared with "During the past 12 months, on how many sports teams run by your school did you play (do not include PE classes)?" for students in grades 8 and 11.

Screen-time behavior was assessed by asking about 1) time spent watching television or video movies away from school; 2) time spent on the computer away from school, surfing the Internet, and instant messaging; and 3) time spent playing video games like Nintendo, Sega, PlayStation, Xbox, Game Boy, or arcade games away from school. Response categories for these questions were 1) I do not watch television or video movies/I do not use the computer/I do not play video games (respectively for each indicator), 2) 1 hour, 3) 2 hours, 4) 3 hours, 5) 4 hours, 6) 5 hours, 7) 6 hours or more. Based on the American Academy of Pediatrics’ recommendation of 2 hours or less of screen-time per day (6), we collapsed these response categories into 2 categories: 2 hours or less per day, and 3 hours or more per day. Lastly, we created a composite variable, sedentary behavior, as the total number of hours spent watching television, using a computer, or playing video games.

### Statistical methods

All estimates and association models used probabilistic sampling weights to account for the multistage sampling design. Given lifestyle and behavioral risk factor differences based on sex, we used linear regression to test mean differences between girls and boys on the number of hours spent watching television, using a computer, or playing video games. We assessed unadjusted differences in demographic characteristics, physical activity, watching television, computer use, playing video games, and sedentary behavior for students' weight status using a multinomial logistic regression model for each sex. We evaluated the combined association of physical activity and watching television on students' weight status using a multinomial logistic regression model for each sex while controlling for age, grade, race/ethnicity, language spoken at home, and percentage of economically disadvantaged students. We reported adjusted odds ratios (AORs) and 95% confidence intervals (CIs). We used Stata version 11.0 (StataCorp LP, College Station, Texas) to calculate estimates, which allowed us to account for the survey sample complex design. Stata uses Taylor linearized methods for variance estimation. Although we evaluated the combined influence of physical activity and computer use as well as physical activity and playing video games on students' weight status, we do not report those results because similar results were observed from the combined influence of physical activity and television viewing. These results are available on request from the authors.

## Results

In 2004-2005, SPAN participants ranged from 8 to 18 years of age. The proportion of girls and boys by racial/ethnic group was similar to the tri-ethnic composition of students in Texas (Table 1). The prevalence of students classified as obese was higher among 4th-grade boys (26%) than 4th-grade girls (21%), whereas the prevalence of students classified as overweight was higher among 8th-grade girls (19%) than among boys in the same grade (17%) (Table 1).

There were significant differences in the proportions of students' weight status by major racial/ethnic groups  and language spoken at home among boys (Table 2). Differences in students' weight status were seen by age and the proportion of economically disadvantaged students among girls as well as among boys (Table 2). Although we observed significant differences in students' age by weight status among girls and boys, those differences were not clinically meaningful. Girls classified as obese had an average age of 12.7 years; boys classified as obese had an average age of 13.1 years. We controlled for these age differences in the multivariate analysis using grade as the proxy for age given that age and grade generated multicollinearity.

Overall, 19% of students were classified as being obese, 75% of students met recommended levels of RPA, 42% of students reported watching 3 or more hours of television per day, and 59% of students had played on at least 1 sports team during the 12 months before the survey. Boys who were classified as obese reported having the highest average number of hours spent watching television, using a computer, or playing video games of all students in this study (Table 2). The mean number of hours spent watching television, using a computer, or playing video games among girls was 4.1 hours per day (95% CI, 3.9-4.3) compared with 5.9 hours per day among boys (95% CI, 5.3-6.0).

Multinomial logistic analysis results establish the combined influence of physical activity and television watching on weight status of students (Table 3). Girls and boys watching low or high levels of television did not have increased odds of being overweight regardless of the level of physical activity (all indicators). Girls who participated in 2 days or less per week of EST and watched 2 hours or less per day of television had increased odds of being obese compared with girls who participated in 3 or more days per week of EST and watched 2 hours or less per day of television. Boys in our study who watched 3 hours or more per day of television had increased odds of being obese, regardless of the level of participation in 3 of 5 physical activity indicators (RPA, PE, and ST). Boys who did not participate in OPA and watched 3 hours or more per day of television had increased odds of being obese compared with boys who participated in OPA and watched 2 or less hours per day of television. Boys who participated in 2 days or less per week of EST and watched 3 hours or more per day of television had increased odds of being obese compared with boys who participated in 3 days or more per week of EST and watched 2 hours or less per day of television.

## Discussion

The independent effects of physical activity and television viewing on the risk of obesity in children and adolescents have been extensively reported (10,11). We observed interaction effects between television watching and physical activity in all of our 5 physical activity indicators among boys. Only the interaction between strengthening exercises and watching television was significant among girls.

Our findings contribute to a limited body of research on the interaction effects of television watching and physical activity with obesity among children and adolescents. Similar to our findings, a report from the 2001 YRBS in the United States found that boys who participated in moderate physical activity on 2 days or less and watched 4 hours or more per day of television had increased odds for being overweight or obese (18). Parsons and colleagues (19) also observed an association between physical activity levels and television viewing related to BMI among 11-year-old girls in a British cohort study. Conversely, Andersen and colleagues (20), in their research on the interaction between daily hours of watching television and weekly sessions of vigorous exercise with BMI among US children and adolescents in 1988 to 1994, found that the interaction was not significant and that television watching was more closely related to BMI.

Because boys in our study who watched more television were more likely to be obese regardless of the amount of activity they reported for physical activity, these data may lend support to the view that television watching influences obesity through advertising and increased snacking rather than displacing physical activity (21). Evaluating whether there is a displacement of physical activity as a result of increased television watching still warrants further research; however, other studies show mixed results (19). Thus, obesity prevention interventions targeting obese boys should address both the need to increase physical activity levels as well as the need to reduce the number of hours per day watching television.

Our findings underscore the continued magnitude of obesity in Texas school children across distinct grade levels. Consistent with previous findings (2), the prevalence of obesity among Texas students in the 2004-2005 academic year among 11th-grade students (23% for boys and 12% for girls) was higher than self-reported obesity levels among same-grade students in the United States in 2003 (17% for boys and 9% for girls), 2005 (17% for boys and 9% for girls), or 2007 (17% for boys and 8% for girls) (22). The population of Texas differs from the general US population in the larger percentage of Hispanic/Latino children and adolescents in Texas. Hispanics tend to have higher rates of obesity than non-Hispanic whites or non-Hispanic blacks (1). During 2004-2005, Texas students reported a higher percentage of participation in RPA (75%) than US high school students reported in 2003 (63%) (22). It is possible that students in Texas were enrolled in physical activities but they did not engage in enough vigorous physical activity. Lastly, the prevalence of Texas students in 2004-2005 who watched 3 or more hours per day of television on an average school day (42.4% for grades 4, 8, and 11) was higher than that observed for US high school students between 2003 and 2007 (range, 35%-38%) (22).

Research is needed to evaluate the effects of the use of new computer gaming systems both during physical education classes at schools and at home. Unfortunately, our question about video game viewing on the questionnaire did not specifically evaluate the use of these new computer gaming systems. A more permanent solution is to institute, finance, and monitor policies that increase minutes of physical activity at school (23) and limit television watching at school or other student settings, such as after-school programs. This will require creating community environments where physical activity is easily accessible and supported (eg, parks, active transportation, and opportunities for individual and team sports). In addition, family policies that place limits on time watching television (24) present promising approaches that should be disseminated to encourage parents and children to decrease television watching at home.

This study has several limitations, most notably the use of self-reported survey data. Although physical activity data were self-reported by the students, which is subject to recall (25) and social desirability (26) biases, these self-reported measures in adolescents have been evaluated for reliability and validity (13,14). The number of hours spent watching television was also self-reported, which may have underestimated the daily number of hours watching television; however, in our study, even with this potential underestimation, we saw an association among boys. We accounted for the variability of the multistage probability-based design by using sampling weights and linearization methods to provide estimates, but there is the potential that cluster effects still remain because a simple randomized selection is logistically difficult and was not implemented.

This study has many strengths: analyses were based on a large and representative sample from an ethnically diverse population with a substantial number of Latino/Hispanic students, direct measurement of heights and weights for students, and evaluation of the combined association between physical activity and watching television. To our knowledge, this is the first study to report the effect of this combined association on our 3 levels of students' weight status (underweight/normal, overweight, and obese) simultaneously using a multinomial model. The advantage of using this model is that estimates are provided for each level and estimated simultaneously in comparison with the reference category (underweight/normal).

## Acknowledgments

The authors thank Jerri Ward and Joey Walker for the coordination of the data collection; Roy Allen for database management; and Kimberley Bandelier, Lesli Beidinger, and Kristy Hanson for assistance with data collection. We also thank the school districts, schools, and children who participated in the study. We acknowledge the assistance of David Christopher Gillis with data management and table preparation and Paige Binder and Raja Malkani for reformatting the manuscript. This study was funded by the Texas Department of State Health Services with funds from the CDC Health and Human Services Block Grant, and the National Institutes of Health, National Institute on Minority Health and Health Disparities, P20MD000170-019001 and P20MD000170-019003, and the Michael and Susan Dell Foundation. The first author (A.P.) was supported by a research supplement (3R37CA057030-20S1) from the National Cancer Institute during the writing of this manuscript.

## Figures and Tables

**Table 1:** Weighted Estimates of Demographic Characteristics of Students by Sex and Grade, School Physical Activity and Nutrition Survey, Texas, 2004-2005^a^

Characteristic	**Girls, **% (95% CI)^a^			**Boys, **% (95% CI)^a^		

	**4th Grade** (n = 3,951)	**8th Grade** (n = 4,499)	**11th Grade** (n = 3,249)	**4th Grade** (n = 3,956)	**8th Grade** (n = 4,328)	**11th Grade** (n = 3,207)
**Major racial/ethnic groups**						
African American	13.4 (10.5-17.0)	14.9 (10.3-20.9)	14.7 (9.7-21.6)	13.5 (8.8-20.1)	14.6 (9.0-22.8)	13.8 (9.0-20.5)
Hispanic	44.7 (37.5-52.1)	41.7 (34.6-49.2)	36.9 (29.9-44.4)	44.5 (38.2-51.0)	41.6 (31.8-52.1)	37.0 (31.4-42.9)
White/other^b^	41.9 (34.3-49.8)	43.4 (36.9-50.2)	48.4 (38.1-58.9)	42.0 (36.6-47.6)	43.8 (33.0-55.2)	49.3 (41.6-57.0)
**Weight status^c^**						
Underweight/normal	60.6 (58.3-62.9)	65.1 (60.6-69.4)	72.0 (67.5-76.2)	55.2 (52.0-58.5)	63.9 (57.3-70.0)	60.9 (54.9-66.6)
Overweight	18.7 (14.4-23.9)	18.7 (16.4-21.3)	16.0 (13.1-19.5)	18.6 (14.8-23.2)	17.4 (13.6-21.9)	16.5 (12.9-20.8)
Obese	20.7 (16.4-25.7)	16.2 (12.8-20.3)	11.9 (8.8-16.1)	26.2 (23.2-29.3)	18.7 (15.3-22.7)	22.6 (19.5-26.1)
**Language spoken at home **						
English	69.3 (59.1-77.9)	74.3 (69.3-78.7)	82.6 (77.0-87.1)	69.0 (58.9-77.6)	75.2 (68.0-81.2)	77.7 (71.7-82.8)
Spanish	25.4 (16.9-36.2)	21.8 (17.5-26.8)	15.7 (11.3-21.3)	25.8 (16.5-37.9)	22.2 (15.6-30.6)	18.1 (14.0-23.1)
Other language	5.3 (3.8-7.4)	3.9 (1.9-8.0)	1.7 (0.9-3.4)	5.2 (2.0-12.6)	2.6 (0.8-7.9)	4.2 (2.1-8.3)
**Age, mean, y**	9.7 (9.6-9.8)	13.7 (13.6-13.8)	16.7 (16.6-16.8)	9.8 (9.7-9.9)	13.8 (13.7-13.8)	16.8 (16.7-16.8)
**Economically disadvantaged students^d ^(mean)**	64.7 (56.9-72.5)	56.9 (49.8-64.0)	42.9 (35.3-50.5)	62.1 (55.7-68.6)	57.2 (49.9-64.6)	44.4 (38.2-50.6)

Abbreviation: CI, confidence interval.

a The n values are the sample size. The total estimated student populations using the sampling weights for girls are 4th grade, N = 121,542; 8th grade, N = 143,561; 11th grade, N = 115,818; and for boys, 4th grade, N = 127,296; 8th grade, 148,111; and 11th grade, N = 117,935.

b White/other category includes non-Hispanic white, Asian, Pacific Islander, Native American, and "other."

c Weight categories by sex and age: underweight, body mass index (BMI) less than the 5th percentile; normal weight, BMI 5th-84th percentile; at risk for overweight, BMI 85th-94th percentile; overweight, BMI 95th or greater percentile.

d Calculated as the sum of the students eligible for free or reduced-price lunch or eligible for other public assistance, divided by the total number of students times 100.

**Table 2:** Demographic Characteristics, Physical Activity, Watching Television, and Screen-Time Behavior by Sex and Weight Status of Students, School Physical Activity and Nutrition Survey, Texas, 2004-2005^a^

Characteristics	Girls, % (95% CI)^b^			Boys, % (95% CI)^b^		

	Underweight/Normal (n = 7,416)	Overweight (n = 2,175)	Obese (n = 2,108)	Underweight/ Normal (n = 6,763)	Overweight (n = 1,976)	Obese (n = 2,752)
**Major racial/ethnic groups**						
African American	13.0 (10.1-16.5)	16.6 (12.6-21.6)	17.4 (12.8-23.3)	13.4 (10.2-17.4)^c^	14.7 (10.6-19.9)^c^	15.0 (10.4-21.3)^c^
Hispanic	39.7 (35.4-44.2)	41.8 (34.6-49.5)	46.6 (42.3-51.0)	36.8 (31.4-42.5)^c^	44.6 (36.3-53.1)^c^	50.4 (44.4-56.4)^c^
White/other^d^	47.3 (41.8-53.0)	41.5 (34.8-48.6)	36.0 (30.2-42.2)	49.9 (42.9-56.8)^c^	40.8 (35.0-46.9)^c^	34.6 (29.3-40.2)^c^
**Language spoken at home**						
English	75.6 (71.0-79.7)	76.6 (70.8-81.6)	72.1 (64.0-78.9)	76.0 (71.6-80.0)^e^	69.9 (62.1-76.7)^e^	71.6 (65.4-77.0)^e^
Spanish	20.1 (16.5-24.2)	21.1 (16.2-26.9)	25.2 (18.3-33.7)	19.3 (15.0-24.5)^e^	25.5 (18.7-33.7)^e^	27.2 (21.7-33.4)^e^
Other language	4.3 (2.8-6.7)	2.3 (1.2-4.4)	2.7 (1.4-5.1)	4.7 (2.8-7.7)^e^	4.6 (1.8-11.4)^e^	1.3 (0.8-2.0)^e^
Met regular physical activity recommendations^f^	72.8 (69.0-76.3)	72.9 (66.9-78.1)	70.3 (65.8-74.4)	78.7 (74.3-82.4)	76.4 (69.5-82.1)	79.1 (75.4-82.4)
Participated in organized physical activities	40.3 (36.8-44.0)	40.5 (33.3-48.2)	33.0 (27.1-39.5)	27.6 (24.6-30.9)	29.9 (23.2-37.5)	23.0 (19.7-26.7)
Participated in exercises to strengthen or tone muscles on 3 days or more of the past 7 days	44.8 (39.3-50.5)	40.0 (32.4-48.1)	38.9 (32.6-45.6)	61.5 (56.7-66.1)	69.0 (58.9-77.6)	58.3 (51.1-65.1)
Participated in physical education classes on 4 or more days during an average school week	41.3 (34.6-48.3)	40.5 (31.3-50.3)	34.3 (28.5-40.7)	45.1 (38.7-51.8)	48.5 (39.7-57.4)	44.5 (36.7-52.5)
**Physical activity/screen-time behaviors**						
Participated in the past year in 1 or more sports teams run by the school	54.2 (49.7-58.7)	61.1 (54.9-66.9)	47.8 (42.2-53.6)	63.2 (58.3-67.7)	69.3 (61.5-76.1)	60.2 (54.1-66.0)
Watched television 3 or more h/d outside school	40.9 (36.9-44.9)	39.5 (34.1-45.2)	42.0 (37.3-46.8)	41.0 (35.5-46.6)	43.0 (37.2-48.9)	52.7 (48.1-57.2)
Spent 3 or more h/d on a computer	15.0 (12.1-18.3)	15.1 (9.2-23.7)	12.7 (9.8-16.3)	16.0 (13.4-18.9)	17.0 (11.5-24.3)	17.8 (13.2-23.4)
Spent 3 or more h/d playing video games away from school	4.3 (3.2-5.9)	5.2 (3.4-8.1)	7.2 (4.3-11.7)	23.4 (19.9-27.2)	26.0 (21.2-31.6)	26.5 (22.1-31.4)
Hours spent watching television, using a computer, or playing video games, mean	4.1 (3.8-4.4)	3.9 (3.6-4.2)	4.1 (3.8-4.4)	5.4 (5.1-5.8)	5.4 (5.0-5.8)	5.9 (5.6-6.3)
**Age, mean, y**	13.5 (12.9-14.1)^g^	13.2 (12.5-13.9)^g^	12.7 (12.1-13.3^g^	13.5 (13.0-14.1)^h^	13.3 (12.7-13.9)^h^	13.1 (12.5-13.7)^h^
**Economically disadvantaged students^i ^**	53.4 (48.4-58.4)^j^	54.8 (49.8-59.8)^j^	62.4 (56.9-67.9)^j^	53.6 (49.2-57.9)^k^	55.3 (50.1-60.4)^k^	58.6 (54.7-62.4)^k^

Abbreviation: CI, confidence interval.

a Weighted percentage and mean estimates with 95% CI.

b The n values are the sample sizes. The total estimated student populations using the sampling weights for underweight girls are N = 250,580; overweight girls, N = 68,105; and obese girls, N = 62,236. For boys, the total estimated student populations using the sampling weights for underweight boys, N = 236,750; overweight boys, 68,893; and obese boys, N = 87,699.

c Boys' global weighted *F* test of unadjusted multinomial logistic model (4,381) = 3.76; *P* = .005.

d White/other category includes non-Hispanic white, Asian, Pacific Islander, Native American, and "other."

e Boys' global weighted *F* test of unadjusted multinomial logistic model (4,381) = 5.01; *P* < .001.

f Defined as engaging in vigorous physical activity that made the respondent sweat and breathe hard for at least 20 minutes on 3 or more of the past 7 days or moderate physical activity that did not cause the respondent to sweat or breathe hard for at least 30 minutes on 5 or more of the past 7 days.

g Girls' global weighted *F* test of unadjusted multinomial logistic model (2,385) = 9.83, *P* < .001.

h Boys' global weighted *F* test of unadjusted multinomial logistic model (2,383) = 3.69, *P* = .026.

i Calculated as the sum of the students eligible for free or reduced price lunch or eligible for other public assistance, divided by the total number of students times 100.

j Girls' global weighted *F* test of unadjusted multinomial logistic model (2,385) = 11.77, *P* <.001.

k Boys' global weighted *F* test of unadjusted multinomial logistic model (2,383) = 5.78, *P* = .003.

**Table 3:** Combined Association of Physical Activity and Watching Television on Weight Status of Students by Sex, School Physical Activity and Nutrition Survey, Texas, 2004-2005^a^

Physical Activity Behaviors	Watched ≤2 h/d of Television Outside School^b^, AOR (95% CI)		Watched ≥3 h/d of Television Outside School^b^, AOR (95% CI)	

	Overweight	Obese	Overweight	Obese
**Girls**				
**Regular physical activity^c^**				
Met recommendations	1 [Reference]	1 [Reference]	0.89 (0.65-1.22)^d^	0.80 (0.57-1.12)
Did not meet recommendations	1.05 (0.74-1.48)	0.92 (0.65-1.31)	0.88 (0.59-1.32)	1.26 (0.78-2.04)^e^
**Organized physical activities**				
Participated	1 [Reference]	1 [Reference]	0.89 (0.58-1.38)	0.80 (0.52-1.24)
Did not participate	1.02 (0.69-1.52)	1.36 (0.97-1.92)	0.90 (0.65-1.24)	1.45 (0.88-2.39)
**Exercises to strengthen or tone muscles**				
Participated ≥3 d/wk	1 [Reference]	1 [Reference]	0.88 (0.48-1.63)	1.13 (0.66-1.96)
Participated ≤2 d/wk	1.46 (0.80-2.64)	1.80 (1.09-2.96)	1.02 (0.62-1.68)	1.03 (0.64-1.65)
**Physical education classes**				
Participated ≥4 d/wk	1 [Reference]	1 [Reference]	0.94 (0.65-1.36)	0.93 (0.61-1.41)
Participated ≤3 d/wk	1.09 (0.76-1.57)	1.30 (0.94-1.81)	0.91 (0.56-1.48)	1.23 (0.89-1.70)
**No. of sports teams participated in at school**				
≥1 team	1 [Reference]	1[Reference]	0.82 (0.53-1.26)	0.77 (0.50-1.17)
No teams	0.72 (0.48-1.08)	1.20 (0.76-1.91)	0.70 (0.49-1.00)	1.36 (0.97-1.91)
**Boys**				
**Regular physical activity^c^**				
Met recommendations	1 [Reference]	1 [Reference]	1.06 (0.76-1.46)	1.61 (1.23-2.09)
Did not meet recommendations	1.10 (0.52-2.35)	0.82 (0.54-1.25)	1.12 (0.62-2.01)	1.40 (1.01-1.94)
**Organized physical activities**				
Participated	1 [Reference]	1 [Reference]	0.68 (0.38-1.21)	1.22 (0.77-1.93)
Did not participate	0.69 (0.42-1.13)	1.06 (0.72-1.56)	0.87 (0.54-1.38)	1.88 (1.26-2.80)
**Exercises to strengthen or tone muscles**				
Participated ≥3 d/wk	1 [Reference]	1 [Reference]	1.01 (0.68-1.50)	1.25 (0.88-1.78)
Participated ≤2 d/wk	0.52 (0.27-1.02)	0.84 (0.52-1.36)	0.94 (0.52-1.71)	1.67 (1.09-2.56)
**Physical education classes**				
Participated ≥4 d/wk	1 [Reference]	1 [Reference]	1.50 (1.00-2.22)	1.72 (1.14-2.60)
Participated ≤3 d/wk	1.11 (0.70-1.75)	0.95 (0.67-1.33)	0.84 (0.54-1.31)	1.50 (1.08-2.09)
**No. of sports teams participated in at school**				
≥1 team	1 [Reference]	1 [Reference]	0.93 (0.69-1.25)	1.45 (1.03-2.03)
No teams	0.62 (0.40-0.95)	0.99 (0.61-1.60)	0.86 (0.52-1.45)	1.92 (1.17-3.17)

Abbreviations: AOR, adjusted odds ratio; CI, confidence interval.

a Statistical significance was set at *P* < .05.

b Estimated from multinomial logistic regression adjusted for age, grade, race/ethnicity, language spoken at home, and percentage of economically disadvantaged students.

c Defined as engaging in vigorous physical activity that made the respondent sweat and breathe hard for at least 20 minutes on 3 or more of the past 7 days, or moderate physical activity that did not make the respondent sweat or breathe hard for at least 30 minutes on 5 or more of the past 7 days.

d This is an example of how to identify the reference category for overweight that applies to all variables in this table. Girls who watched TV for 3 or more hours per day outside school and met recommendations for regular physical activity have an adjusted odds ratio of 0.89 for being overweight (95% CI, 0.65-1.22) compared with normal-weight girls who watched TV 2 or less hours per day outside school and met recommendations for regular physical activity.

e This is an example of how to identify the reference category for obesity that applies to all variables in this table. Girls who watched TV for 3 or more hours per day outside school and did not meet recommendations for regular physical activity have an adjusted odds ratio of 1.26 for being obese (95% CI, 0.78-2.04) compared with normal-weight girls who watched TV for 2 or less hours per day outside school and met recommendations for regular physical activity.
